# Prevalence and determinants of home delivery among reproductive age women, Margibi County, Liberia

**DOI:** 10.1186/s12884-022-04975-7

**Published:** 2022-08-19

**Authors:** Leroy S. Maximore, Abdul Gafaru Mohammed, Gyesi Razak Issahaku, Samuel Sackey, Ernest Kenu

**Affiliations:** 1 Ghana Field Epidemiology & Laboratory Training Program, Accra, Ghana; 2 Department of Epidemiology and Disease Control, School of Public Health, University of Ghana, Accra, Ghana; 3 Ministry of Health, Paynesville, Liberia; 4 National Public Health Institute, Paynesville, Liberia; 5 Tamale Teaching Hospital, Tamale, Ghana

**Keywords:** Home delivery, Institutional delivery, Margibi County, Reproductive age, Liberia

## Abstract

**Background:**

The use of institutional delivery services is essential for improving maternal and child health. However, studies in Liberia reveal over 20% of women still deliver at home. We assessed the prevalence and associated factors of home delivery among women of reproductive age in Margibi County, Liberia.

**Methods:**

We conducted a cross-sectional study among 438 women of reproductive age in Margibi County. Data were obtained using a semi-structured questionnaire. A simple random sampling approach was used to select the participants for the study. We performed binary logistic regression to identify factors influencing home delivery. Findings were summarized into tables displaying the frequencies, percentages, crude, and adjusted odds ratios (ORs) and 95% confidence intervals (CIs).

**Results:**

Prevalence of home delivery in the County was 90.6% (95% CI = 87.5 – 93.0). Women who were ≥ 31 years (aOR = 6.74, 95%CI = 2.86—15.90), women who had two or more children (aOR = 9.68, 95%CI = 4.07—22.99) and those who had rapid onset of labor (aOR = 6.35, 95%CI = 1.59 – 25.27) were associated with increased odds of home delivery. Good attitude of health workers (aOR = 0.01, 95%CI = 0.001 – 0.08) and the availability of transport to the nearest health facility (aOR = 0.01, 95%CI = 0.003 – 0.03) were factors associated with a decreased odds of home delivery among the study participants.

**Conclusion:**

The high prevalence of home delivery in the county is a call for urgent interventions by the government of Liberia and various non-governmental organizations. The government may need to supply the county with ambulances and ensure in-service training of health workers on good attitudes.

**Supplementary Information:**

The online version contains supplementary material available at 10.1186/s12884-022-04975-7.

## Background

Pregnancy and childbirth have been documented as a period of increased vulnerability in almost all societies and throughout history, during which mothers and babies need help, especially from skilled birth attendants or midwives [1]. Home delivery is ingrained in our country’s unique history, and was our forebears’ preferred way of childbirth. The amount of births attended by professional health workers is one of the two criteria for measuring progress towards achieving Millennium Development Goal (MDG) 5 [2]. As one of the measures to enhance maternal and neonatal survival, global policies seek to move the place of delivery from home to health facility [3, 4]. This has led to significant rises in facility delivery [5].

In developing countries, a large proportion of mothers still deliver at home compared to developed countries [68.7% as against 1.3%] [6]. In Liberia, home delivery continues to be relatively high compared to developed countries, 44% and 20% in 2013 and 2019 respectively [7–9]. While Liberia has embraced the World Health Organization’s policy of prohibiting deliveries at home, 56% of the deliveries in rural communities are undertaken at home, of which Margibi county contributes a significant amount [7–9]. In Margibi County, health facility delivery is less than 80%, with an estimated over 85% of maternal mortality attributed to home delivery [10]. Infant mortality and under-five mortality rate in the County have been estimated at 65 per 1000 and 111 per 1000 live births respectively [10].

To increase health facility delivery in Liberia, the Government implemented the free delivery and maternal health care policy as part of the post-conflict National Health and Social Welfare Policy and Plan (NHSWPP) in all public health facilities in 2011 [11]. Despite the free delivery policy, home delivery remains high [8]. In this context, it is vital to comprehend specific factors that influence women’s decision to give birth at home and analyse any differences to improve services. Various factors have been reported to influence home delivery among pregnant women. These include; poor quality health services, low educational level, health care cost, socio-cultural practices, maternal parity and knowledge on pregnancy risk factors [12, 13]. An increase in home deliveries may likely lead to high maternal and newborn morbidities and mortalities due to complications related to the delivery [14].

Although comprehensive studies have been conducted on home delivery in other countries across Africa and Asia, there is still a literature gap since many of the studies are undertaken outside Liberia [15–22]. This study is conducted to better understand why home delivery remains very high in the Margibi county of Liberia, and to identify associated factors that can help inform interventions that may reduce the prevalence of home delivery in this county.

## Methods and population

### Study design and period

We conducted a community-based cross-sectional survey among 438 reproductive-age women who gave birth between June 2020 and May 2021 in Margibi County. The survey included all four health districts in the county. Data was collected using a semi-structured questionnaire. The research participants were distributed among the four districts based on the proportion of registered reproductive-age women who gave birth between June 2020 and May 2021.

### Study area

We conducted the study in Margibi County, Liberia. The county is subdivided into four health districts, with an estimated population of 269,570. The annual delivery rate is 4%, and women of reproductive age were 62,001 for the 2019—2020 fiscal year [10]. According to the Margibi Developmental Agenda (MDA), over 42% of the population resides in the urban area, where more health facilities and ambulance services are available [23]. The county has 62 health facilities, of which two are Hospitals, 14 Health Centers and 46 Clinics. Twenty-four (24) of the 62 health facilities are government-owned. The county has one government referral hospital located in the Kakata district which has the majority of the skilled and qualified public health workforce. The Margibi County Health Team manages the county's health sector with technical and financial support from the central Ministry of Health and partners. The county has two functional ambulances for the referral of cases from one level to another. The county transport system is accessible only in urban communities, while rural communities are difficult to reach.

### Study population and eligibility

The study population comprised women of reproductive age 15 – 49 years who gave birth at least once a year preceding the survey and resided in the County for more than six months. Only the most recent delivery was considered among women with more than one live birth in the preceding year. Critically sick women or those who could not communicate were excluded from the study.

### Sample size determination and sampling procedure

The sample size was determined using Cochran formula [24]. Assuming a power of 80%, at a 95% confidence interval and a prevalence of 52% from a study in Ethiopia [25] with a precision of 5% and a 14% non-response rate, we had a minimum sample size of 438. A proportionate allocation method was used to determine the number of participants from each district in the Margibi county based on the number of registered reproductive-age women who gave birth between June 2020 and May 2021, obtained from each district health office. The number of registered women who gave birth included; Firestone District (1199), Gibi District (1069), Kakata District (2566) and Mambah-Kaba District (1401). Following proportionate allocation, reproductive-age women who fulfill the inclusion criteria were selected randomly from the list at each district.

### Data collection tool, procedure and quality control

We used a semi-structured questionnaire and performed face-to-face interviews with study participants in their various communities to collect data. The developed questionnaire was pre-tested in Grand Bassa County among 25 randomly sampled mothers. Fifteen midwives in the county were recruited and trained for three (3) days to collect data. The midwives were trained on all components of the semi-structured questionnaire, consenting process, sampling of participants, safety of participants and researchers, privacy and confidentiality in the data collection process. The questionnaire was prepared in English and translated into the local languages spoken by the participants: Bassa, Kpelle, Kisi and Gbandi. The questionnaire was in three sections; sociodemographic characteristics, institutional factors and community-level factors. The data collected included; age, occupation, educational status, marital status, gravidity, parity, number of antenatal visits, distance from community to health facility, nature of labor, attitude of health workers and the availability to and from the health facilities. We observed all COVID-19 protocols before conducting the interviews. The principal investigator conducted data cleaning and cross-checking to ensure accuracy and consistency.

### Data management and statistical analysis

Data collected were cleaned in Microsoft Excel 2018 and exported into Stata version 16 for statistical analysis. Categorical variables were summarized into frequencies and percentages at a 95% Confidence interval. Parameter estimates were reported as percentages with their corresponding 95% confidence interval. A binary logistic regression analysis was used to determine the presence of an association between home delivery and each independent variable. Variables that have shown a *P*-value of 0.2 or less were selected and fitted into multivariable logistic regression for controlling possible confounders. Variables with *P*-value less than 0.05 were considered statistically significant. Prior to performing the adjusted logistic regression, a multicollinearity test using the variance inflation factor and goodness-of-fit test (Hosmer and Lemeshow model fitness test) was performed to determine the model fitness.

## Results

### Characteristics of study participants and prevalence of home delivery, Margibi County, Liberia

Out of the 438 respondents surveyed, 159 (36.3%) were residents of the Kakata district. The majority, 232 (53.0%) of the respondents, were above 31 years. The majority, 325 (74.2), of the respondents indicated health workers at the various health facilities had poor attitudes toward clients. On their most recent delivery, the majority 284 (64.8) indicated they gave birth during the rainy season (Table 1).

**Table 1:** Characteristics of the study respondents and prevalence of home delivery, Margibi County, Liberia

Characteristics	n (%)	Prevalence (95% CI)
**Place of delivery**		
Health facility	41 (9.4)	
Home	397 (90.6)	90.6 (87.5 93.2)
**District**		
Firestone	104 (23.7)	88.5 (80.7 93.9)
Gibi	50 (11.4)	88.0 (75.7 95.5)
Kakata	159 (36.3)	89.3 (83.4 93.6)
Mamba Kabah	125 (28.5)	95.2 (89.8 98.2)
**Age**		
< 31 years	206 (47.03)	83.5 (77.7 88.3)
≥ 31 years	232 (52.97)	97.0 (93.9 98.8)
**Marital Status**		
Single	52 (11.9)	86.5 (74.2 94.4)
Married	386 (88.1)	91.2 (87.9 93.8)
**Education**		
No formal education	310 (70.8)	89.4 (85.0 93.0)
Primary	103 (23.5)	93.2 (86.0 97.0)
Secondary or higher	25 (5.7)	96.0 (80.0 100)
**Onset of labor**		
Slow	134 (30.6)	74.6 (66.4 81.7)
Rapid	304 (69.4)	97.7 (95.3 99.1)
**Season/period in the year**		
Dry season	154 (35.2)	77.9 (70.5 84.2)
Rainy season	284 (64.8)	97.5 (95.0 99.0)
**Gravida**		
Primigravid	55 (12.6)	87.3 (75.5 94.7)
Multigravida	383 (87.4)	91.1 (87.8 93.8)
**Parity**		
Primiparous	75 (17.1)	64.0 (52.1 74.8)
Multiparous	363 (82.8)	96.1 (93.6 97.9)
**Attitude of staff **^c^		
Poor	325 (74.2)	87.7 (83.6 91.1)
Good	113 (25.8)	99.1 (95.2 100.0)
**Type of Setting**		
Rural	409 (93.4)	90.2 (86.9 92.9)
Urban	29 (6.62)	96.6 (82.2 99.9)
**Transport** ^a^		
No	386 (88.13)	98.2 (96.3 99.3)
Yes	52 (11.87)	34.6 (22.0 49.1)
**Male Health worker **^b^		
No	361 (82.42)	95.8 (93.2 97.7)
Yes	77 (17.58)	66.2 (54.6 76.6)
**Religion**		
Christianity	406 (92.69)	90.1 (86.8 92.9)
Islam	32 (7.31)	96.8 (83.8 99.9)

^a ^Transport: Access to transport at the time of delivery

^b ^Male health worker: General preference/aversion to male health workers

^c ^Attitude of staff: Perceived attitude of health workers

The overall prevalence of home delivery for participants’ most recent child in the county was 90.6% (95% CI**:** 87.5—93.2). High prevalence of home delivery was recorded among multiparous women 96.1% (95% CI: 93.6—97.9) than primiparous women 64.0% (95% CI: 52.1—74.8). Higher prevalence of home delivery 98.2% (95% CI: 96.3—99.3) was reported among women who have no available transport compared to their counterparts (Table 1).

### Factors associated with home delivery, in Margibi County, Liberia

The district of residence, age, marital status, religion, gravida, parity, season/period in the year, type of setting, the onset of labor, education, male health worker and transport to health facility at the time of delivery were selected for a multivariable logistic regression at *P* < 0.2. From these factors, the age, attitude of health workers, parity, availability of transport to health facility at the time of delivery and onset of labor were significantly associated with home delivery in the county. Women who were ≥ 31 years had 6.7 times increased home delivery odds than women < 31 years (aOR = 6.74, 95%CI = 2.86—15.90). Women who had two or more children had 9.7 times the odds of home delivery compared to primiparas (AOR = 9.68, 95%CI = 4.07—22.99). Women who had a rapid onset of labor had 6.4 times odds of home delivery compared to the counterparts (aOR = 6.35 (1.59—25.27). In terms of respondents’ perceived attitude of health workers at the health facility, women who rated health workers' attitudes as good had 99.0% reduced odds of home delivery compared to those who rated health workers' attitudes as poor (aOR = 0.01, 95%CI = 0.001 – 0.08). Women who had means of transport available had 99.0% decreased odds of home delivery compared to their counterparts (aOR = 0.01, 95%CI = 0.003 – 0.03) (Table 2).

**Table 2:** Factors associated with home delivery in Margibi County, Liberia

Characteristics	Crude OR (95% CI)	Adjusted OR (95% CI)	*P*-value
**District**			
Firestone	1	1	
Gibi	0.96 (0.34 2.72)	0.86 (0.26 2.81)	0.772
Kakata	1.09 (0.50 2.39)	1.13 (0.47 2.74)	0.959
Mamba Kabah	2.59 (0.94 7.15)	3.46 (1.16 10.36)	0.036
**Age**			
< 31 years	1	1	
≥ 31 years	6.35 (2.75 14.68)	6.74 (2.86 15.90)	0.001
**Marital Status**			
Single	1	1	
Married	0.62 (0.26 1.48)	0.75 (0.30 1.87)	0.312
**Education**			
Elementary	1	1	
Secondary or higher	1.75 (0.21 14.91)	2.56 (0.26 25.14)	0.199
No formal education	0.61 (0.26 1.43)	0.89 (0.34 2.32)	0.296
**Onset of labor**			
Slow	1	1	
Rapid	14.43 (6.20 33.56)	6.35 (1.59 25.27)	0.001
**Season/period in the year**			
Dry season	1	1	
Rainy season	11.21 (4.83 26.00)	3.44 (0.86 13.71)	0.241
**Gravida**			
Primigravid	1	1	
Multigravida	1.50 (0.63 3.56)	0.69 (0.28 1.71)	0.113
**Parity**			
Primiparous	1	1	
Multiparous	14.02 (6.88 28.59)	9.68 (4.07 22.99)	0.001
**Attitude of staff**			
Poor	1	1	
Good	0.06 (0.01 0.45)	0.01 (0.001 0.08)	0.001
**Type of Setting**			
Rural	1	1	
Urban	3.04 (0.40 22.91)	8.67 (0.84 89.72)	0.115
**Transport**			
No	1	1	
Yes	0.01 (0.004 0.03)	0.01 (0.003 0.03)	0.001
**Male Health worker**			
No	1	1	
Yes	0.09 (0.04 0.17)	0.68 (0.21 2.18)	0.532
**Religion**			
Christianity	1	1	
Islam	3.39 (0.45 25.49)	9.54 (0.91 100.34)	0.167

## Discussion

Pregnant women's choice of place of delivery is usually an important decision at the final stage of their gestational period. Some of these pregnant women deliver at home, whereas some go to the health facility. Despite the numerous interventions by the WHO and programmes implemented by various governments of Liberia, home delivery remains one of the significant challenges the country faces. The current study revealed that more than 90% of the women studied delivered at home in their most recent delivery in Margibi County.

The high prevalence rate recorded is consistent with the findings of a study conducted in Zala Woreda, southern Ethiopia, where 77% of the women studied reported delivering at home in their most recent delivery [26]. In a similar study conducted in the Dodota district of Northwest Ethiopia, almost 80% of the women studied reported delivering at home [27]. The high prevalence of home delivery among pregnant women is further substantiated by another study conducted in Akure, Nigeria, where 81.8% of women studied mentioned delivering at home in their most recent delivery [28]. However, in Mukono District-Uganda and Jimma Zone, Southwest Ethiopia, the prevalence level of home delivery was less than 35% [25, 29]. Unlike these studies, our study considered women who delivered within one year prior to the study as our inclusion criteria. This could have accounted for the inconsistency in the prevalence level recorded. To increase health facility delivery, one of the measures the government of Liberia can adopt is providing either monetary or non-monetary incentives to pregnant women or women who deliver at a health facility. This approach has been proven beneficial by various countries that adopted the approach in the past. The adopted Janani Suraksha Yojna’ (JSY) by India where pregnant women are provided with cash incentives to encourage ANC attendance and health facility delivery has been reported to increase health facility delivery by 46.2% [30]. Similarly, in a study conducted in Kenya, nurses believed the provision of incentives encouraged pregnant women to deliver at the health facility [31].

The factors associated with home delivery among pregnant women in the county were age, parity, the attitude of health workers, the onset of labor and availability of transport.

Women who had given birth more than once were more likely to deliver at home compared to women who gave birth for the first time. This finding is similar to the findings of other studies done in Southwest Ethiopia, Trincomalee, Sri Lanka and Nepal [25, 32, 33]. This might be because women who have given birth several times perceive themselves to be more experienced in labor, thereby developing more interest in using home delivery services [34, 35].

The attitude of health workers towards clients who access health care services is instrumental to the care-seeking participation rate. Pregnant women who perceive health workers to be of good attitude were more likely to deliver at a health facility. This finding is consistent with the results of a study conducted in Uganda, where women who rated health workers' attitudes as poor had 5.4 times increased odds of home delivery. Similarly, in a survey conducted in Bahirdar, Ethiopia, pregnant women who rated health workers' attitudes as poor had 4.4 times increased odds of home delivery compared to their counterparts. In a study involving women in the Sekela district of West Ethiopia, the odds of home delivery was 6.0 times increased among those who perceive health workers to be of poor attitude compared to those who perceive them to be of good attitude [32, 34, 36, 37]. Resources should be channeled into attitude training of these health workers and supportive supervision to ensure they act accordingly.

The availability of transport at the time of delivery is another factor associated with home delivery in this study. The county is faced with limited health facilities with people travelling long distances from the rural areas to access health care services in the urban areas. Pregnant women in labor who do not have readily available means of transport to a health facility are likely to give birth at home. This is consistent with the findings of a study conducted in Zambia where limited availability and cost of transport were associated with an increased rate of home delivery [38]. Nurses should educate pregnant women on the need to make arrangements for transport using their estimated expected date of delivery.

The problem of recall bias was a limitation in this study. Women were required to recall their past experiences in their last childbirth which could be as long as 12 months prior to the survey. Research assistants reviewed the women's ANC cards to support their responses to curtail this limitation. Also, the vast majority of women in the study were from rural areas, which does not reflect the overall urban–rural distribution of the population.

## Conclusion

The prevalence of home delivery in the county was over 90%. Women more than 31 years, multiparous women, and rapid onset of labor were significantly associated with increased odds of home delivery. The availability of transport and the good attitude of health workers were associated with reduced odds of home delivery. At a policy level, we recommend that the MOH conduct in-service training for healthcare providers on positive attitudes towards patients. The Government of Liberia should make available ambulance vehicles in all county districts and provide incentives for health facility delivery.

## Supplementary Information

**Additional file 1.**

## Data Availability

The datasets used and/or analyzed during the current study are available from the corresponding author on reasonable request.

## Abbreviations

ANC: Antenatal Care
MDGs: Millennium Development Goals
WHO: World Health Organization
NHSWPP: National Health and Social Welfare Policy and Plan
