# Supplementary material for: Prevalence and determinants of home delivery among reproductive age women, Margibi County, Liberia
Source: BMC Pregnancy Childbirth. 2022 Aug 19;22:653. doi: 10.1186/s12884-022-04975-7 (PMC9389515; doi:10.1186/s12884-022-04975-7)
Supplement: Supplementary file 1 — Additional file 1. [file 12884_2022_4975_MOESM1_ESM.docx]

Appendix I: Questionnaire

**Study on Factors influencing home delivery amongst pregnant women, in Margibi County, Liberia,**

Hi, my name is Leroy S. Maximore, a student at the University of Ghana and conducting a study on **Factors influencing home delivery amongst pregnant women in Margibi County, Liberia,**

Findings from this study will be used by the Margibi County Health Team (MaCHT) and policymakers to establish reasonable steps in minimizing home deliveries and enhancing maternal health and pregnancy outcomes. Are you willing to participate in our study? ________________

| Serial number_______________ | Date_____________________ |
| --- | --- |
| Start time__________________ | End time__________________ |
| Longitude__________________ | Latitude ___________________ |

| **SECTION A: DEMOGRAPHY / INDIVIDUAL FACTORS** | | |
| --- | --- | --- |
| 1 | District of resident | 1. Firestone ( ) b. Gibi ( ) c. Kakata ( ) d. Mamba Kabah ( ) |
| 2 | Community/ Town/ Village |  |
| 3 | Participant’s age | 1. ≤ 14 ( ) b. 15-20 ( ) c. 21-30 ( ) d. 31-40 ( ) e. 41-49 ( ) |
| 4 | Mother's Occupation: | 1. Farmer ( ) b. Trader ( ) c. Housewife ( ) d. Tapper e. Other ( ) specify_________________________________ |
| 5 | Husband's Occupation: | a. Farmer ( ) b. Tapper ( ) c. Trader ( ) d. Other ( ) specify_____________________ |
| 6 | Marital Status: | 1. Single ( ) b. Cohabiting ( ) c. Married () d. Divorced ( ) e. Widowed ( ) f. Separated ( ) g. Other, specify_______________ |
| 7 | Highest level of Education? | a. no formal education () b. Elementary ( ) c. Junior High School ( ) d. Senior High School ( ) e. Tertiary ( ) |
| 8 | Husband's highest level of Education? | a. No formal education () b. Elementary ( ) c. Junior High School ( ) d. Senior High School ( ) e. Tertiary ( ) |
| 9 | Which Religion do you worship? | a. Christianity ( ) b. Islam ( ) c. Traditional ( ) d Other ( ), specify _____________ |
| 10 | Average monthly Income | 1. US ≤ $50 ( ) b. US ≥ $50 ( ) |
| 11 | Will you allow a male health worker to deliver you? | 1. Yes ( ) b. No ( ) |

| **SECTION B: DELIVERY HISTORY /OUTCOME** | | | | | | |
| --- | --- | --- | --- | --- | --- | --- |
| 1 | | How many deliveries have you had? |  | | | |
| 2 | | How many children are alive now? |  | | | |
| Where did you deliver them? (list for each delivery and outcome of each delivery) | | | | | | |
| 11a | Did you receive Antenatal Care? | | | Yes | No | I don't know |
| 11b | If yes, where? a. Health facility ( ) b. Traditional Maternity home ( ) c. Other ( ) specify___________________ | | | | | |
| 12 | At what month of pregnancy did you go for first Antenatal visit? ___________ | | | | | |
| 13 | How many Antenatal Care visits did you have altogether during the last pregnancy _________________ | | | | | |
| 14 | Where did you deliver your most recent child? a. Health facility ( ) b. Home ( ) | | | | | |

| SECTION C: COMMUNITY FACTORS | | | | |
| --- | --- | --- | --- | --- |
| 1 | | Do you seek permission before you visit the health centre during pregnancy and delivery? | a. Yes () b. No () | |
| 1a. | | If Yes, from whom? |  | |
| 2 | | In your family structure who decides the place of delivery when you are pregnant? | a. Myself ( ) b. My in-laws ( ) c. My husband ( ) d. The gods ( )  e. Other ( ), specify_____________ | |
| 3 | | If you choose to deliver in a health facility what will be your husband's response? | a. He will agree ( ) b. Culture does not allow ( ) c. He will not agree ( ) d. There will be doubt ( ) | |
| 4 | | Do you have any traditional norm or law in your area/ community that prevent you from delivering at home? | 1. Yes ( ) b. No ( ) | |
| 5 | | Is there any community action or sanction against those who deliver at home | a. Yes ( ) b. No ( ) | |
| 6 | | If yes, what sanction is it? | a._________________ | |
| 7 | | Does distance from your community to the health facility a problem to a facility delivery? | a. Yes ( ) b. No ( ) | |
| **SECTION D**: **INSTITUTIONAL/ HEALTH FACILITY FACTORS** | | | | |
| 1 | Cost of health facility delivery | | | a. Cheap ( )  b. Moderate ( )  c. High ( )  d. Very high ( ) |
| 2 | Availability of skilled personnel? | | | a. Yes ( )  b. No ( ) |
| 3 | Attitude of staff | | | a. Very poor ( )  b. Poor ( )  c. Good ( )  d. Very good ( ) |
| 4 | In your community, what do people usually say about health facility delivery services? | | | a. Expensive ( )  b. No midwife ( )  c. No drugs ( )  d. Staff not friendly ( )  e. Not expensive ( ) |
| 5 | Why is it that some women deliver in health facilities, but others do not? | | | a. Means of transport ( )  b. Have insurance card ( )  c. Poverty ( )  d. Cost of services ( ) |

| PHYSICAL ACCESSIBILITY | | |
| --- | --- | --- |
| 1 | Distance from community to health facility | a. ≥ 5 Km ( )  b.≤ 5Km ( ) |
| 2 | Availability of transport at the time of delivery | 1. Yes ( ) 2. b. No ( ) |
| 3 | Is health facility delivery affordable | 1. Yes () 2. b. No ( ) |
| 4 | Season/period in the year you delivered | 1. Rainy season ( ) 2. Dry season ( ) |
| 5 | A rural or urban setting | 1. Rural ( ) 2. b. Urban ( ) |
| 6 | Onset of labor | 1. Yes ( ) 2. b. No ( ) |
